# Psychological capital and organizational citizenship behavior among nurses during the COVID-19 epidemic: mediation of organizational commitment

**DOI:** 10.1186/s12912-023-01332-7

**Published:** 2023-05-19

**Authors:** Li Zeng, Fen Feng, Man Jin, Wanqing Xie, Xin Li, Lan Li, Yihang Peng, Jialin Wang

**Affiliations:** 1 Sichuan Nursing Vocational College, No.173 Longdu South Road, Longquanyi District, Chengdu City, 610100 Sichuan province China; 2 College of Nursing, Chengdu University of Traditional Chinese Medicine, No.1166 Liutai Road, Wenjiang District, Chengdu City, 611137 Sichuan province China; 3 Chengdu University of Traditional Chinese Medicine Affiliated Hospital, No.39 Shierqiao Road, Jinniu District, Chengdu City, 610072 Sichuan province China; 4 The Third People’s Hospital of Chengdu, No.82 Qinglong Street, Qingyang District, Chengdu City, 610014 Sichuan province China; 5 West China Dental Hospital of Sichuan University, No. 14 section 3, Renmin South Road, Wuhou District, Chengdu City, 610000 Sichuan province China; 6 Army Medical Center of PLA, No. 10 Changjiang Branch Road, Daping, Yuzhong District, Chongqing City, 400042 China

**Keywords:** Nurses, Psychological capital, Organizational commitment, Organizational citizenship behavior, COVID-19

## Abstract

**Background:**

Nurses’ organizational citizenship behavior, a spontaneous “altruistic work behavior”, may be affected by psychological capital and organizational commitment, but its mechanism is not clear. The aim of this study was to investigate the characteristics and distribution of psychological capital, organizational commitment and organizational citizenship behavior among nurses during the COVID-19 epidemic, and explore the mediating role of organizational commitment in psychological capital and organizational citizenship behavior.

**Methods:**

A cross-sectional survey was conducted among 746 nurses from 6 designated hospitals for COVID-19 treatment in China. Descriptive statistics, Pearson correlation analysis, and structural equation model were used in this study.

**Results:**

Nurses’ psychological capital, organizational commitment and organizational citizenship behavior scores were 103.12 ± 15.57, 46.53 ± 7.14 and 101.47 ± 12.14, respectively. Additionally, organizational commitment partially mediates between psychological capital and organizational citizenship behavior.

**Conclusions:**

During the COVID-19 pandemic, nurses’ psychological capital, organizational commitment, and organizational citizenship behavior were found to be at an upper-middle level, influenced by various social-demographic factors. Furthermore, the results illustrated that psychological capital can affect organizational citizenship behavior through the mediating role of organizational commitment. Therefore, the findings emphasize the importance of nursing administration to monitor and prioritize the mental health and organizational behavior of nurses during the ongoing COVID-19 crisis. It is crucial to focus on developing and nurturing nurses’ psychological capital, strengthening their organizational commitment, and ultimately promoting their organizational citizenship behavior.

## Background

The COVID-19 pandemic, a contagious disease of great magnitude, has presented unprecedented challenges to global medical and healthcare systems [1]. As the country most affected by COVID-19 in its early stages and on the broadest scale, China has long entered the normalization phase of epidemic prevention and control. However, the physical and psychological trauma to which people have been subjected is incalculable, which has led many scholars to conduct a range of surveys and studies targeting different populations. Despite extensive research, current evidence suggests that there is no definitive treatment for this disease, and therapeutic interventions mainly rely on a combination of antiviral agents and supportive care [2, 3]. Nurses, the main caregivers who provide supportive care for patients with COVID-19, often face a complex and demanding work environment, resulting in significant work intensity and pressure, which may affect nurses’ “altruistic work behavior”, such as organizational citizenship behavior [1].

Organizational citizenship behavior amongst nurses is spontaneous behavior that is not necessarily mandated by the formal salary system or organizational requirements, but which serves to sustain and augment the social and psychological environment of the organization and drive the attainment of task goals [4]. Research indicated that nurses’ organizational citizenship behavior was linked to their psychological capital and organizational commitment, ultimately impacting nursing organizational performance [5, 6]. The job demands-resources (JD-R) model also pointed out that personal resources (such as psychological capital) can stimulate workers’ motivation (such as organizational commitment), which has a positive impact on employees’ job performance (such as organizational citizenship behavior) [7].

While a significant amount of research has shown that psychological capital and organizational commitment play a positive role in promoting nurses’ organizational citizenship behavior, few studies have delved into the specifics of how this relationship functions, especially in the context of the COVID-19 pandemic. To enable nursing managers to better understand how to promote nurses’ organizational citizenship behavior and enhance overall nursing performance during the COVID-19 pandemic, it is crucial to elucidate the connections between nurses’ psychological capital, organizational commitment, and organizational citizenship behavior.

### Psychological capital

Nurses’ psychological capital refers to the positive psychological energy exhibited by nurses in their personal growth and development, which manifests as a state-like motivation tendency [8]. This valuable resource can be leveraged through targeted investment and development efforts to gain a competitive advantage. According to the conservation of resources (COR) theory, psychological capital is a critical personal resource that enhances the availability of psychological resources, reduces vulnerability to resource loss, and promotes positive feedback such as high-level organizational commitment and work engagement [9, 10]. Additionally, research has found that nurses with high levels of psychological capital not only proactively complete their own tasks but also help colleagues with non-required tasks, demonstrating organizational citizenship behavior [11].

### Organizational commitment

Organizational commitment refers to the extent to which employees recognize and embrace the goals and values established by their organization, feel emotionally attached to the organization, and are willing to remain and make meaningful contributions to it over time [12, 13]. Research conducted by Kazemipour et al. (2012) and Taghinezhad et al. (2015) have both highlighted that organizational commitment has a positive impact on nurses’ job satisfaction, care behaviors, self-perceived job performance, and likelihood to exhibit organizational citizenship behavior [5, 14].

Based on the aforementioned information, it is evident that there exists a close relationship between nurses’ psychological capital, organizational commitment, and organizational citizenship behavior. However, research on the interplay and connections among these factors, especially in the context of the ongoing COVID-19 pandemic, remains limited. Thus, drawing on the JD-R model, the primary aim of this study is to examine the associations between nurses’ psychological capital, organizational commitment, and organizational citizenship behavior during the COVID-19 epidemic. Furthermore, this study also seeks to explore the mediating impact of organizational commitment on the relationship between psychological capital and organizational citizenship behavior, and the goal is to establish a strong theoretical basis for boosting nurses’ work motivation, enhancing nursing organizational performance, and promoting the morale and productivity of nurses during the COVID-19 pandemic. The research hypotheses are as follows:

#### Hypothesis 1

Nurses’ psychological capital is positively correlated with organizational citizenship behavior during the COVID-19 pandemic.

#### Hypothesis 2

Nurses’ organizational commitment is positively correlated with organizational citizenship behavior during the COVID-19 pandemic.

#### Hypothesis 3

Nurses’ organizational commitment plays a mediating role between psychological capital and organizational citizenship behavior during the COVID-19 pandemic.

## Methods

### Aims and design

This is a descriptive, cross-sectional study employing a path analysis approach to determine the mediating effect of nurses’ organizational commitment in the relationship between psychological capital and organizational citizenship behavior during the COVID-19 epidemic.

### Data collection and sample

From January 2021 to May 2021, convenient sampling was used to select clinical nurses from 6 designated hospitals for COVID-19 treatment in Chengdu, China. We first contacted the hospital administrator and obtained permission for investigation. The survey was mainly conducted through the network platform, and each hospital had a volunteer responsible for the investigation feedback and follow-up work of the hospital, and another research assistant monitored the network platform at any time during the survey. The electronic questionnaire included a unified guide to introduce the purpose, variables and filling requirements, and pointed out that it was deemed informed consent to fill in without omission and submit the questionnaire, and the survey was anonymous and voluntary. If there were problems with the quality of the questionnaire, the research assistant would quickly contact the volunteer and asked him to help strengthen the communication with the respondents to ensure the reliability of the data. A total of 800 questionnaires were distributed and 746 valid questionnaires were recovered, with an effective recovery rate of 93.25%.

The participants work in different departments, including internal medicine, surgery, obstetrics, pediatrics and infection department, etc. The inclusion criteria were as follows: (a) to have obtained the nurse certificate; (b) to be engaged in clinical nursing for ≥ 1 year; (c) informed consent and voluntary participation in this study. The exclusion criteria were as follows: (a) informal staff, such as refresher nurses; (b) nurses who were not on duty during the investigation; (c) nurses suffering from major physical diseases or mental disorders during the investigation; (d) nurses who experienced major family changes during the survey.

### Ethical consideration

This study has been approved by the Ethics Committee of Chengdu University of Traditional Chinese Medicine (No. 2020-KL084).

### Measures

#### Social-demographic information questionnaire

The social-demographic information questionnaire included gender, age, education level, marital status, years of work experience, work hours per day, sleep hours per day, monthly income and participation in mental health-related training, with a total of 9 items.

#### Psychological capital Questionnaire-24 (PCQ-24)

PCQ-24 compiled by Luthans et al. (2007), includes four dimensions of self-efficacy, hope, resiliency and optimism, with a total of 24 items, including three reverse scoring questions, items 13, 20 and 23 respectively [8]. Participants were asked to answer with a 6-point Likert scale, ranging from “strongly disagree” to “strongly agree”, score 1–6 points respectively, and the total score was the sum of the scores of each item. The higher the score, the higher the level of psychological capital. Through literature review, it was found that PCQ-24 had been proved to have good reliability and validity in Chinese nurse population [10]. In this study, the Cronbach’s α reliability value of PCQ-24 was 0.939. The KMO value of 0.811 and the Bartlett’s test yielded a significant chi-square value of 2011.611 (*p* < 0.001), indicating suitability for factor analysis. The confirmatory factor analysis (CFA) showed a good fit for the model (*χ2/df* = 2.498, GFI = 0.942, AGFI = 0.921, NFI = 0.958, CFI = 0.974, TLI = 0.967 and RMSEA = 0.045), suggesting that PCQ-24 demonstrates good reliability and validity in the present sample of the study.

#### Chinese version Organizational Commitment Questionnaire (C-OCQ)

The original version of OCQ was developed by Chang et al. (2009) to measure nurses’ organizational commitment level, including three dimensions: value commitment, effort commitment and retention commitment, with a total of 12 items. C-OCQ translated by Cheng et al. (2017), and participants were asked to answer with a 5-point Likert scale, ranging from “strongly disagree” to “strongly agree”, they received 1–5 points respectively [12, 13]. The higher the score, the higher the level of organizational commitment. It was found that C-OCQ had been proved to have good reliability and validity in Chinese nurse population, and score of 12–24 indicated a low level of organizational commitment, a score of 25–47 indicated a medium level, and a score of 48–60 indicated a high level [15]. In this study, the Cronbach’s α reliability value of C-OCQ was 0. 949. The KMO value of 0.705 and the Bartlett’s test yielded a significant chi-square value of 1583.962 (*p* < 0.001), indicating suitability for factor analysis. The confirmatory factor analysis (CFA) showed a good fit for the model (*χ2/df* = 4.396, GFI = 0.964, AGFI = 0.926, NFI = 0.981, CFI = 0.985, TLI = 0.974, RMSEA = 0.068), suggesting that C-OCQ demonstrates good reliability and validity in the present sample of the study.

#### Nurses’ organizational citizenship behavior scale (NOCBS)

NOCBS compiled by Wan et al. (2015), includes five dimensions: self-development, responsibility consciousness, actively serving, helping colleagues, and organizational identity, with a total of 24 items [16]. Participants were asked to answer with a 5-point Likert scale, ranging from “very inconsistent” to “very consistent”, they received 1–5 points respectively. It was found that NOCBS had been proved to have good reliability and validity in Chinese nurse population, and if the average score of items reached 3, it was a medium level, and the higher the score, the more organizational citizenship behavior of nurses [17]. In this study, the Cronbach’s α reliability value of NOCBS was 0. 951. The KMO value of 0.876 and the Bartlett’s test yielded a significant chi-square value of 4165.749 (*p* < 0.001), indicating suitability for factor analysis. The confirmatory factor analysis (CFA) showed a good fit for the model (*χ2/df* = 3.721, GFI = 0.919, AGFI = 0.883, NFI = 0.966, CFI = 0.975, TLI = 0.967, RMSEA = 0.060), suggesting that NOCBS demonstrates good reliability and validity in the present sample of the study.

### Statistical analysis

SPSS version 23.0, AMOS version 23.0 were used to analyze the data. Since the data were all from self-report, it was likely to produce common method biases and reduce the research validity. Therefore, Harman’s single-factor test was used to analyze the common method biases of the questionnaire data [18]. Cronbach’s α coefficient, KMO, Bartlett’s test and CFA were used to test the reliability and validity of PCQ-24, C-OCQ and NOCBS. Descriptive analysis, independent sample T-test and one-way analysis of variance (ANOVA) were used to describe and compare the scores of organizational citizenship behavior of nurses with different social-demographic information. Pearson correlation analysis was used to determine whether there was a correlation between nurses’ psychological capital, organizational commitment and organizational citizenship behavior. Structural equation model was used to explore the mediating role of organizational commitment between psychological capital and organizational citizenship behavior. The *χ*^*2*^/df < 5, Tacker-Lewis index (TLI), comparative fit index (CFI), incremental fit index (IFI), relative fit index (RFI) and normal fit index (NFI) > 0.90, root mean square error of approximation (RMSEA) ≤ 0.08 are considered to be reasonable model fitting. When testing the significance of mediation effect, the number of repeated sampling was set to 5000 and the confidence interval was set to 95%, and when the confidence interval of each path coefficient does not contain 0, it indicated that the mediation effect was significant [19]. In this study, *p* < 0.05 was considered statistically significant (two-tailed test).

## Results

### Preliminary analysis

To examine the common method biases in the questionnaire data, we applied Harman’s single-factor test. The unrotated exploratory factor analysis resulted in 14 factors, with the first factor explaining 31.890% of the variance. However, as this was below the critical threshold of 40%, it suggested that the data in this study were not subject to severe common method biases [18].

### Participant characteristics

Among the 746 participants, 4.69% were males and 95.31% were females, and their average age was 31.23 ± 6.51, average years of nursing experience was 9.38 ± 7.07, average work hours per day was 8.60 ± 1.58, and average sleep hours per day was 6.79 ± 0.85. More than half of the nurses were married (67.56%), had bachelor degree or above (75.87%) and had not participated in mental health-related training (56.70%). The number of nurses with a monthly income (CNY) of 4000–6000 was the largest (36.19%) (Table 1).

**Table 1:** Demographic characteristics, and the distribution of organizational citizenship behavior (N = 746)

Variables	N (%)	Mean (SD)	*t/F*	*p*
Gender			0.037	0.847
Male	35 (4.69)	101.09 (9.47)		
Female	711 (95.31)	101.49 (12.26)		
Age (years)			7.502	0.000
≤ 25	147 (19.70)	98.74 (12.67)		
26–35	452 (60.59)	101.17 (11.88)		
36–45	110 (14.75)	104.61 (11.67)		
≥ 45	37 (4.96)	106.70 (11.29)		
Education level			0.002	0.967
Associate degree or less	180 (24.13)	101.51 (12.85)		
Bachelor degree or above	566 (75.87)	101.46 (11.92)		
Marital status			7.447	0.001
Unmarried	226 (30.30)	98.96 (12.56)		
Married	504 (67.56)	102.49 (11.83)		
Divorced/Separated	16 (2.14)	105.06 (10.91)		
Years of nursing experience			5.630	0.000
≤ 5 years	238 (31.90)	99.13 (12.21)		
5 to 10 years	274 (36.73)	101.22 (12.13)		
11 to 15 years	123 (16.49)	103.70 (12.09)		
16 to 20 years	56 (7.51)	103.48 (11.07)		
> 20 years	55 (7.37)	105.89 (10.99)		
Work hours per day			6.530	0.011
≤ 8 h	519 (69.57)	102.22 (12.28)		
> 8 h	227 (30.43)	99.76 (11.66)		
Sleep hours per day			8.423	0.004
≤ 7 h	588 (78.82)	100.81 (12.23)		
> 7 h	158 (21.18)	103.95 (11.52)		
Monthly income (CNY)			3.154	0.014
≤ 4000	106 (14.21)	99.73 (12.89)		
4001–6000	270 (36.19)	101.26 (11.69)		
6001–8000	209 (28.02)	101.31 (12.67)		
8001–10,000	112 (15.01)	101.53 (11.25)		
> 10,000	49 (6.57)	107.00 (11.49)		
Participation in mental health-related training			5.314	0.001
Not participated	423 (56.70)	100.01 (12.49)		
Once a year	165 (22.12)	102.63 (11.84)		
2 times / year	92 (12.33)	103.95 (10.76)		
≥ 3 times / year	66 (8.85)	104.53 (11.18)		

### The factors associated with organizational citizenship behavior

Independent-samples T-test and one-way ANOVA revealed that nurses who were aged 45 or higher, more than 20 years of nursing experience, and monthly income (CNY) > 10,000 had higher organizational citizenship behavior scores (*p* < 0.05), while nurses who were unmarried, worked more than 8 h per day, slept less than 7 h per day, and did not participate in mental health-related training had lower organizational citizenship behavior scores (*p* < 0.05) (Table 1).

Multiple linear regression analyses showed that age (*β* = 0.179, *p* < 0.001), participation in mental health related training (*β* = 0.142, *p* < 0.001), sleep hours per day (*β* = 0.105, *p* = 0.003) and work hours per day (*β* = -0.079, *p* = 0.027) were influencing factors of nurses’ organizational citizenship behavior. Other variables, such as marital status, were not significant in the regression equation (Table 2).

**Table 2:** Multiple linear regression analyses of organizational citizenship behavior (N = 746)

Model	*B*	SE	*β*	*t*	*p*
(Constant)	95.315	1.153		82.671	0.000
Age	2.952	0.586	0.179	5.039	0.000
Participation in mental health-related training	1.752	0.436	0.142	4.015	0.000
Sleep hours per day	3.109	1.059	0.105	2.936	0.003
Work hours per day	-2.084	0.940	-0.079	-2.218	0.027

*F* = 13.705, *p* = 0.000, *R*^2^ = 0.069, Adjusted *R*^*2*^ = 0.064.

### Correlation analyses

Table 3 showed that the participants’ psychological capital, organizational commitment and organizational citizenship behavior scores were 103.12 ± 15.57, 46.53 ± 7.14 and 101.47 ± 12.14, while their average score of items were 4.29 ± 0.65, 3.88 ± 0.60 and 4.23 ± 0.51, respectively. Psychological capital was positively correlated with organizational commitment and organizational citizenship behavior (*r* = 0.636, *p* < 0.001; *r* = 0.503, *p* < 0.001), while organizational commitment was positively correlated with organizational citizenship behavior (*r* = 0.498, *p* < 0.001).

**Table 3:** Means, standard deviations, and correlations of psychological capital, organizational commitment and organizational citizenship behavior (N = 746)

	1	2	3	4	5	6	7	8
Mean	103.12	46.53	101.47	21.14	25.62	20.77	17.26	16.67
SD	15.57	7.14	12.14	2.66	3.22	2.83	2.16	2.28
1. Psychological capital	-							
2. Organizational commitment	0.636^**^	-						
3. Organizational citizenship behavior	0.503^**^	0.498^**^						
4. Self-development	0.455^**^	0.430^**^	0.917^**^					
5. Responsibility consciousness	0.461^**^	0.443^**^	0.952^**^	0.883^**^				
6. Actively serving	0.490^**^	0.527^**^	0.931^**^	0.786^**^	0.847^**^			
7. Helping colleagues	0.411^**^	0.431^**^	0.921^**^	0.797^**^	0.867^**^	0.822^**^		
8. Organizational identity	0.499^**^	0.464^**^	0.884^**^	0.740^**^	0.759^**^	0.828^**^	0.786^**^	

^**^p < 0.001

### Mediation analyses

**Fig. 1:**
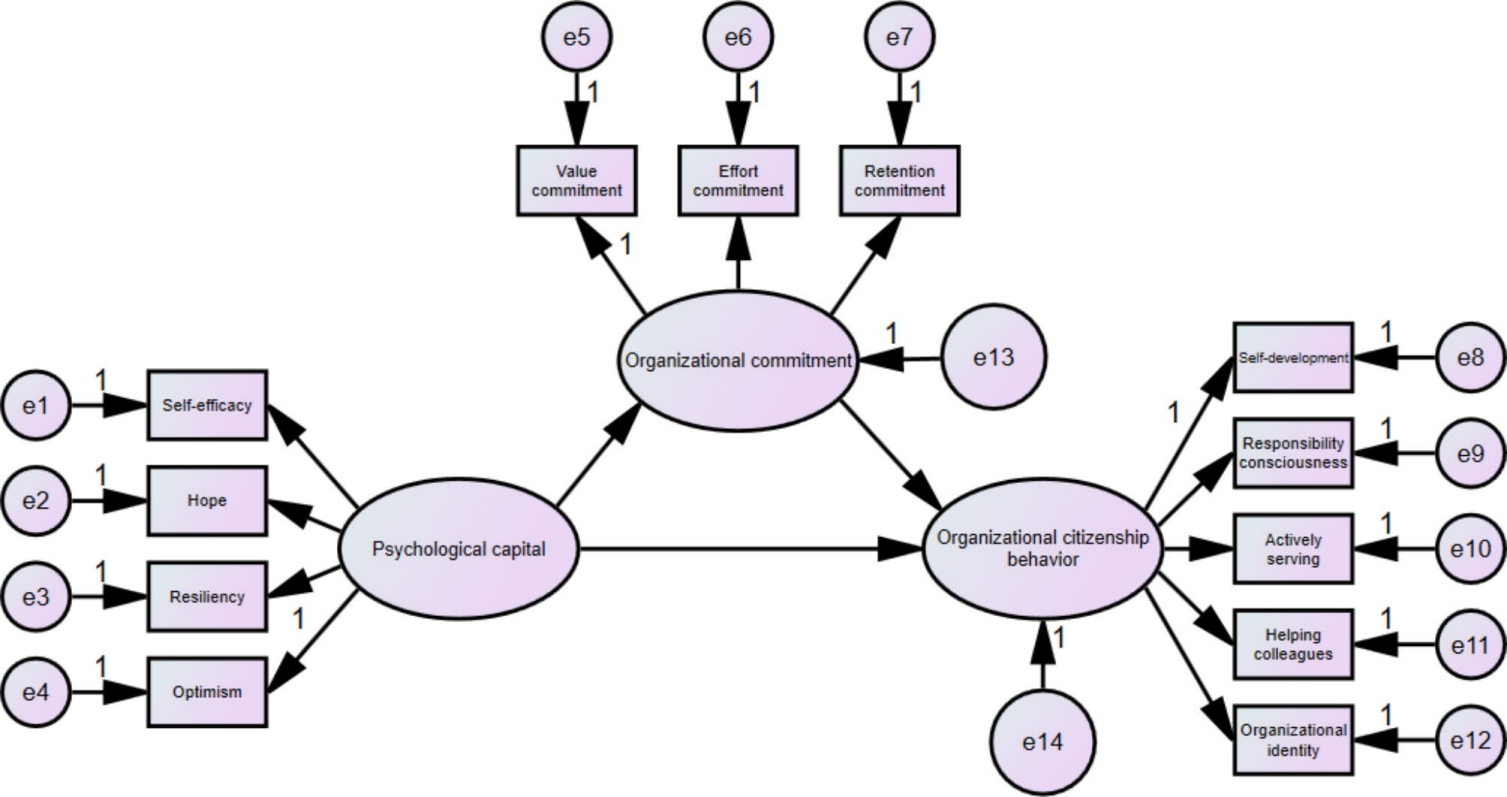
The structural model

The structural model included three latent constructs (psychological capital, organizational commitment and organizational citizenship behavior) and twelve observed variables (Fig. 1). The fit indices indicated that the model was appropriate: *χ*^*2*^/df = 4.731, TLI = 0.972, CFI = 0.981, IFI = 0.982, RFI = 0.964, NFI = 0.977, RMSEA = 0.071. Furthermore, all factor loads of indicators on latent constructs were significant (*p* < 0.05), indicating that all latent constructs were well represented by their indicators.

As shown in Table 4, psychological capital had a significant direct effect on organizational commitment (*β* = 0.624, *p* = 0.001) and organizational citizenship behavior (*β* = 0.339, *p* = 0.001). The direct effect of organizational commitment on organizational citizenship behavior was 0.234 (*p* = 0.001). The indirect effect of psychological capital →organizational commitment →organizational citizenship behavior was 0.146 (*p* = 0.001), which suggested that organizational commitment partially mediates between psychological capital and organizational citizenship behavior.

**Table 4:** Direct and indirect effects for the model (N = 746)

Model pathways	Standardized effect (*β*)	*95% CI*	*p*
Direct effect			
Psychological capital → organizational commitment	0.624	(0.572, 0.674)	0.001
Organizational commitment → organizational citizenship behavior	0.234	(0.145, 0.324)	0.001
Psychological capital → organizational citizenship behavior	0.339	(0.249, 0.428)	0.001
Indirect effect			
Psychological capital → organizational commitment → organizational citizenship behavior	0.146	(0.089, 0.204)	0.001

## Discussion

In this study, we revealed that nurses’ psychological capital, organizational commitment and organizational citizenship behavior were at an upper-middle level during the COVID-19 epidemic. Compared to prior research, the psychological capital reported in this study was similar, but both organizational commitment and organizational citizenship behavior were lower. This observation may be attributed to the complex nursing situations and immense work pressures inherent in providing care within designated hospitals for COVID-19 treatment [4, 15, 20]. Our study suggested that older nurses and those who participated in mental health-related training more frequently reported higher organizational citizenship behavior, whereas those who slept less than 7 h per day and worked over 8 h per day indicated lower organizational citizenship behavior. Jin et al. (2022) posited that as nurses gain more experience, undertake psychological education and training, and start incorporating overall medical care quality and hospital development goals with their own values and objectives, they develop an active willingness to contribute to the hospital, ultimately resulting in voluntary dedication behavior [4]. Additionally, some studies found that long working hours and insufficient sleep time can lead to an imbalance between work and life, causing job burnout and hindering nurses’ growth and development, which eventually may reduce organizational citizenship behavior [21–23]. These results lead us to believe that the psychological well-being and organizational behavior of nurses may have been influenced to some extent by the COVID-19 pandemic. Therefore, we may be able to reduce the impact by increasing attention to the mental health of nurses, especially those with less experience, providing training on mental health-related topics, scheduling work hours reasonably, and encouraging nurses to develop healthy lifestyles.

Hypothesis 1 proposes a positive correlation between psychological capital and organizational citizenship behavior. The results of this study confirmed a positive effect of psychological capital on organizational citizenship behavior, which is consistent with the findings of Bogler et al. (2019) and Lee et al. (2020) [6, 24]. Psychological capital, as a positive personal resource, offers unlimited advantages and tremendous potential, and is crucial for maintaining the mental well-being of nurses and managing work-related stressors [25]. Moreover, the higher the level of psychological capital among nurses, the greater their sense of workplace happiness, which in turn encourages voluntary behavior to improve work efficiency and nursing service quality, ultimately promoting patient recovery, enhancing overall organizational performance, and endowing medical and nursing organizations with a unique competitive advantage [26, 27]. Furthermore, in this study, the correlation coefficient between the psychological capital and organizational citizenship behavior of nurses surveyed during the COVID-19 pandemic was higher than the results obtained during non-pandemic periods, indicating a closer relationship between the two variables [24]. This finding suggests that in order to promote “altruistic work behavior” and improve organizational performance among nurses during the COVID-19 pandemic, attention must be paid to their individual levels of psychological capital.

The results obtained regarding the relationship between organizational commitment and organizational citizenship behavior allowed us to corroborate Hypothesis 2, in line with the studies carried out by Cho & Kao (2022) [28]. Nurses with higher levels of organizational commitment are more likely to see a connection between their own efforts and the achievement of organizational goals, which further strengthens this connection as their level of commitment gradually increases. As a result, these nurses may internalize the organization’s goals and take actions to achieve them, leading to more organizational citizenship behaviors [29, 30]. This study not only confirmed the link between organizational commitment and organizational citizenship behavior among nurses during the COVID-19 pandemic but our results also showed a higher correlation coefficient between the two variables than in previous studies [29]. This suggests that in the context of the pandemic, it is even more important to foster emotional connections between nurses and their organizations, to focus nurses on their nursing duties, and to cultivate their sense of dedication, ultimately prompting them to exhibit more organizational citizenship behaviors.

Besides, Hypothesis 3 on the mediating effect of organizational commitment had also been confirmed by the research. In the context of the COVID-19 pandemic, positive psychological capital can increase the level of organizational commitment among nurses, strengthening their recognition of the goals and values set by the organization, fostering greater sense of identification and belonging towards the organization, enhancing their willingness to work for and stay with the organization. This, in turn, leads to more active focus on work, the willingness to complete additional tasks, and exhibit high levels of organizational citizenship behavior [10, 30, 31]. Our findings highlight that during the COVID-19 pandemic, the impact of nurses’ psychological capital on their organizational citizenship behavior is not simply a direct effect, but also an indirect effect involving organizational commitment as a mediating variable. This reflects the internal mechanism connecting nurses’ psychological and organizational behaviors in the specific context and provides insight for managers to better understand and address nurses’ psychological state and work behavior, facilitating the allocation of resources to promote overall performance improvement.

Finally, the direct impact of psychological capital on organizational citizenship behavior can be explained by the COR theory. In essence, individuals with higher levels of psychological capital have access to more psychological resources, which can be readily invested to acquire additional reserves. By doing so, they are better equipped to generate organizational citizenship behavior and achieve successful career development [6, 9, 32]. Moreover, the indirect effects of organizational commitment as mediating variables can be explained by the JD-R model. As a valuable personal resource, psychological capital can stimulate individual work motivation and foster a stronger sense of organizational commitment. These factors, in turn, contribute to the development of organizational citizenship behavior, as identified by Bogler et al. (2019), Tang et al. (2019), and Cho & Kao (2022) [24, 28, 31].

### Limitations

Although our research has certain limitations, it is undeniable that the results of this study can be used as an extension of JD-R model in the field of nursing, and this study also has certain theoretical guiding significance for the research direction of nurse psychology and management in the future. The first limitation is that this study adopts a cross-sectional survey design, which means the dynamic changes and potential relationships among nurses’ psychological capital, organizational commitment, and organizational citizenship behavior over time cannot be measured. Secondly, the use of self-report scales in this study may undermine the reliability of the results to some extent. Lastly, convenience sampling is used to select samples, which might impact the universality of the research outcomes.

## Conclusions

Based on the JD-R model, this study explores for the first time the relationship between nurses’ psychological capital, organizational commitment, and organizational citizenship behavior during the COVID-19 pandemic. The study finds that nurses’ psychological capital, organizational commitment, and organizational citizenship behavior are all at an upper-middle level during the pandemic, which is influenced by factors such as age, mental health-related training, sleep hours, and work hours. Furthermore, the study shows that the correlation between nurses’ psychological capital, organizational commitment, and organizational citizenship behavior is higher during the pandemic than in non-pandemic times, and that organizational commitment plays a mediating role between psychological capital and organizational citizenship behavior. In conclusion, measures can be taken to promote nurses’ organizational citizenship behavior. On the one hand, nurses should focus on their mental health, adjust their mindset, adapt to the environment, enhance trust and loyalty to the organization, actively participate in work and activities, and contribute to the improvement of personal and organizational performance. On the other hand, nursing managers should recognize that nurses’ organizational citizenship behavior can be further enhanced by developing and tapping into their psychological capital and increasing their level of organizational commitment.

## Data Availability

The data that support the finding of this study are available from the corresponding author upon reasonable request.

## References

[1] Zhang H, Zhao Y, Zou P, Lin S, Mu S, Deng Q, Gan L (2021). Explaining Organizational Citizenship Behavior among Chinese Nurses combating COVID-19. Risk Manag Healthc Policy.

[2] Tang D, Comish P, Kang R (2020). The hallmarks of COVID-19 disease. PLoS Pathog.

[3] Lyu H, Yao M, Zhang D, Liu X (2020). The Relationship among Organizational Identity, Psychological Resilience and Work Engagement of the First-Line Nurses in the Prevention and Control of COVID-19 based on structural equation Model. Risk Manag Healthc Policy.

[4] Jin M, Zhang Y, Wang F, Huang J, Feng F, Gong S, Wang J (2022). A cross sectional study of the impact of psychological capital on organisational citizenship behaviour among nurses: mediating effect of work engagement. J Nurs Manag.

[5] Taghinezhad F, Safavi M, Raiesifar A, Yahyavi SH (2015). Antecedents of organizational citizenship behavior among iranian nurses: a multicenter study. BMC Res Notes.

[6] Lee SN, Kim JA (2020). Prediction model for nursing work outcome of nurses: focused on positive psychological capital. J Korean Acad Nurs.

[7] Bakker AB, Demerouti E (2017). Job demands-resources theory: taking stock and looking forward. J Occup Health Psychol.

[8] Luthans F, Avolio BJ, Avey JB, Norman SM (2007). Positive psychological capital: measurement and relationship with performance and satisfaction. Pers Psychol.

[9] Hobfoll SE (2002). Social and psychological resources and adaptation. Rev Gen Psychol.

[10] Zhou J, Yang Y, Qiu X, Yang X, Pan H, Ban B, Wang W (2018). Serial multiple mediation of organizational commitment and job burnout in the relationship between psychological capital and anxiety in chinese female nurses: a cross-sectional questionnaire survey. Int J Nurs Stud.

[11] Hu P, Wu Z (2019). The influence of psychological capital of medical staff on organizational citizenship behavior. J Social Sci Nanjing Univ Traditional Chin Med.

[12] Chang CS, Chang HC (2009). Perceptions of internal marketing and organizational commitment by nurses. J Adv Nurs.

[13] Cheng B, Hou YH, Wu QX, Shi YX, Di HJ (2017). Introduction and evaluation of the reliability and validity of chinese version organizational commitment questionnaire. Chin Nurs Manage.

[14] Kazemipour F, Mohamad AS, Pourseidi B (2012). Relationship between workplace spirituality and organizational citizenship behavior among nurses through mediation of affective organizational commitment. J Nurs Scholarsh.

[15] Tai CL, Pang D, Sui SJ, Chen D, Li XY (2021). The mediating role of self-regulatory fatigue between paternalistic leadership and organizational commitment. J Nurs Adm.

[16] Wan QQ, Wang L, Xu CJ, Zhang TH (2015). Validity and reliability of nurses’ organizational citizenship behavior scale. Chin Nurs Manage.

[17] Du L, Peng MQ, Wang AH (2020). A study on the relationship of job embeddedness and organizational citizenship behavior of pediatric nurses. J Nurs Adm.

[18] Podsakoff PM, MacKenzie SB, Podsakoff NP (2012). Source of method bias in social science research and recommendations on how to control it. Ann Rev Psychol.

[19] Hayes AF (2013). Introduction to mediation, moderation, and conditional process analysis: a regression-based approach.

[20] Liu ZY, Wei WH, Wang L, Cui HZ, Li YY, Zhang FL (2017). The relationship among job satisfaction, work engagement and organizational citizenship behavior of nurses. Chi J Behav Med & Brain Sci.

[21] Dall’Ora C, Griffiths P, Ball J, Simon M, Aiken LH (2015). Association of 12 h shifts and nurses’ job satisfaction, burnout and intention to leave: findings from a cross-sectional study of 12 european countries. BMJ Open.

[22] Stimpfel AW, Fletcher J, Kovner CT (2019). A comparison of scheduling, work hours, overtime, and work preferences across four cohorts of newly licensed registered nurses. J Adv Nurs.

[23] Zeng L, Zhang X, Wang F, Yun J, Lai L, Jin M, Wang J (2022). Prevalence and influencing factors of posttraumatic growth among nurses suffering from workplace violence: a cross-sectional study. Int J Ment Health Nurs.

[24] Bogler R, Somech A (2019). Psychological capital, Team Resources and Organizational Citizenship Behavior. J Psychol.

[25] Mubarak N, Safdar S, Faiz S, Khan J, Jaafar M (2021). Impact of public health education on undue fear of COVID-19 among nurses: the mediating role of psychological capital. Int J Ment Health Nurs.

[26] Elliott R, Fry M (2021). Psychological capital, well-being, and patient safety attitudes of nurses and midwives: a cross-sectional survey. Nurs Health Sci.

[27] Yuan Z, Zhang X, Wang F, Jin M, Teng M, He H, Wang J (2023). Levels of psychological capital among nurses: a systematic review and meta-analysis. Int Nurs Rev.

[28] Cho CC, Kao RH (2022). Developing sustainable workplace through leadership: perspectives of transformational leadership and of organizational citizenship behavior. Front Psychol.

[29] Nohe C, Hertel G (2017). Transformational Leadership and Organizational Citizenship Behavior: a Meta-Analytic Test of underlying mechanisms. Front Psychol.

[30] Firmansyah A, Junaedi I, Kistyanto A, Azzuhri M (2022). The effect of perceived organizational support on organizational citizenship behavior and organizational commitment in public health center during COVID-19 pandemic. Front Psychol.

[31] Tang Y, Shao YF, Chen YJ (2019). Assessing the mediation mechanism of job satisfaction and organizational commitment on innovative behavior: the perspective of Psychological Capital. Front Psychol.

[32] Coetzee SK, Laschinger H (2018). Toward a comprehensive, theoretical model of compassion fatigue: an integrative literature review. Nurs Health Sci.

