# Correction: Psychological capital and organizational citizenship behavior among nurses during the COVID-19 epidemic: mediation of organizational commitment

**DOI:** 10.1186/s12912-023-01680-4

**Published:** 2024-01-11

**Authors:** Li Zeng, Fen Feng, Man Jin, Wanqing Xie, Xin Li, Lan Li, Yihang Peng, Jialin Wang

**Affiliations:** 1 Sichuan Nursing Vocational College, No.173 Longdu South Road, Longquanyi District, 610100 Chengdu City, Sichuan province China; 2 College of Nursing, Chengdu University of Traditional Chinese Medicine, No.1166 Liutai Road, Wenjiang District, 611137 Chengdu City, Sichuan province China; 3 Chengdu University of Traditional Chinese Medicine Affiliated Hospital, No.39 Shierqiao Road, Jinniu District, 610072 Chengdu City, Sichuan province China; 4 The Third People’s Hospital of Chengdu, No.82 Qinglong Street, Qingyang District, 610014 Chengdu City, Sichuan province China; 5 West China Dental Hospital of Sichuan University, No. 14 section 3, Renmin South Road, Wuhou District, 610000 Chengdu City, Sichuan province China; 6 Army Medical Center of PLA, No. 10 Changjiang Branch Road, Daping, Yuzhong District, 400042 Chongqing City, China

**Correction to: BMC Nursing (2023) 22:172**

10.1186/s12912-023-01332-7

Following publication of the original article [1], the authors reported that the affiliations of the author Li Zeng should be one and two instead of one. The author affiliations have been updated.

The original article [1] has been corrected.
